# Longitudinal evaluation of peripheral photoreceptor atrophy in fundus albipunctatus

**DOI:** 10.1007/s10384-026-01337-0

**Published:** 2026-03-28

**Authors:** Takuhiro Hayakawa, Kei Mizobuchi, Takaaki Hayashi, Taro Kominami, Koji M Nishiguchi, Shinji Ueno, Kazuki Kuniyoshi, Mineo Kondo, Takeshi Iwata, Kaoru Fujinami, Kazushige Tsunoda

**Affiliations:** 1 Division of Vision Research, National Institute of Sensory Organs, NHO Tokyo Medical Center, 2-5-1 Higashigaoka, Meguro-ku, Tokyo, 152-8902 Japan; 2 Department of Ophthalmology, The Jikei University School of Medicine, Tokyo, Japan; 3 Department of Ophthalmology, Nagoya University Graduate School of Medicine, Aichi, Japan; 4 Department of Ophthalmology, Hirosaki University Graduate School of Medicine, Aomori, Japan; 5 Department of Ophthalmology, Kindai University Faculty of Medicine, Osaka-sayama, Japan; 6 Department of Ophthalmology, Mie University Graduate School of Medicine, Mie, Japan; 7 Division of Molecular and Cellular Biology, National Institute of Sensory Organs, NHO Tokyo Medical Center, Tokyo, Japan

**Keywords:** Fundus albipunctatus, Dots/flecks, Longitudinal observation, OCT, *RDH5*

## Abstract

**Purpose:**

To investigate the long-term longitudinal changes of the retinal morphology and visual function in patients with fundus albipunctatus (FA).

**Study design:**

Retrospective observational study.

**Methods:**

Seventeen Japanese patients with FA who had pathogenic variants of the *RDH5* gene were studied in a multicenter retrospective study.

**Results:**

The baseline ages ranged from 3 to 61 years, and the patients were followed longitudinally for 3 to 21 years. The relative size of the flecks/dots decreased throughout the course of the disease; rapidly in the first and second decades of life and slowly thereafter. The flecks/dots in the OCT images were seen as hyperreflective flat deposits on the retinal pigment epithelium in the first decade of life, and as hyperreflective pile-like formations that extended to the ellipsoid zone (EZ) of the photoreceptors after the second decade of life. At later ages, the EZ in the mid-peripheral retina was not present due to photoreceptor atrophy. The Goldmann visual fields had a peripheral constriction in 7 of 11 cases that developed during the course of the disease process.

**Conclusion:**

The long-term longitudinal observations of the morphological changes of the retina and visual fields revealed that FA is a slowly progressive retinal dystrophy with atrophy of the peripheral photoreceptors in older patients. These findings will be very important information for the patients when providing genetic counseling.

## Introduction

Fundus albipunctatus (FA; OMIM #136880) is a type of stationary night blindness characterized by impaired night vision, and with many small and discrete white dots scattered throughout the retina except in the macula region [1, 2]. FA is caused by biallelic variants of the retinol dehydrogenase 5-encoding gene (*RDH5;* OMIM *601617)[3, 4] The *RDH5* gene is essential for the conversion of 11-cis-retinol to 11-cis-retinal, and dysfunction of this gene leads to a disturbance of the visual cycle with slow recovery of dark-adaptation. FA can be accurately diagnosed by full-field electroretinography (ERG): the rod responses are non-recordable after 20 minutes of dark-adaptation and recover to normal amplitudes following 180 min of dark-adaptation. Several studies show that FA is not necessarily a stationary disorder, and cases with progressive macular atrophy have been described [5, 6]. It is also reported that cone dysfunction was present in the late stages in more than one-half of FA cases [5–8]. Although the pathological mechanisms of FA have not been fully determined, not only cone but also rod photoreceptors in the peripheral retina are expected to be susceptible to dysfunction or atrophy. This is because the *RDH5* gene plays a significant role in the rod visual cycle. However, there has not been any systematic investigations of the morphology and function of the peripheral retina in FA patients.

The purpose of this study was to investigate the long-term morphological and functional changes of the retina of patients with FA to determine whether the peripheral retina is preserved in the later stages of FA.

## Subjects and methods

Informed consent was obtained from all subjects after an explanation of the procedures to be used for the examinations was presented. Permission was also obtained to use their medical data for research.

The procedures used adhered to the tenets of the Declaration of Helsinki, and approval to perform this study was obtained from the Review Board/Ethics Committee of the NHO Tokyo Medical Center (Reference: R18-029), the Jikei University School of Medicine (Reference; 24–231 6997), Nagoya University Graduate School of Medicine (Reference; 2020-0598), Kindai University Faculty of Medicine (Reference; 22-132 and R05-071), and Mie University Graduate School of Medicine (Reference; MIE-2429).

We searched for Japanese patients with FA carrying pathogenic variants of the *RDH5* gene in the Genotype-Phenotype Database of the Japan Eye Genetics Consortium (JEGC; Table 1) [9]. We reviewed the clinical records of the patients whose fundus photographic and OCT data were available for the analyses. In the end, we analysed the clinical data of 17 patients (10 men and 7 women), who visited one of the 5 collaborating institutions between 2003 and 2024. The age at the baseline ranged from 3 to 61 years, and the patients had been followed longitudinally for 3 to 21 years. Part of the genotype-phenotype correlations of this cohort were presented in our earlier report.[8]

**Table 1:** Clinical characteristics and genetic findings in patients

Patient No."	Sex	Chief complaint	Age at baseline	BCVA at baseline (Decimal)		Age at final visit	BCVA at final visit (Decimal)		Morphological changes of white flecks/dots during follow-up over 5 years	Peripheral constriction and/or midperipheral /paracentral blind spot of Goldmann VF at the final visit	Macular degeneration	Variants in *RDH5* gene (NM_001199771.3)
				OD	OS		OD	OS				
1 (KA042)	F	Night blindness	55	1.0	1.0	68	1.0	1.0	Fading and disappearance of dots.	+	+	c.927_928insGAA, p.928C>G, :p.L310delinsEV (homo)
2 (KA394)	M	Night blindness	5	1.0	1.0	17	1.0	1.0	Changes in location and size of flecks. Conversion from large flecks to small dots.	(-)	(-)	c.927_928insGAA, p.928C>G, :p.L310delinsEV (homo)
3 (KA441)	M	Night blindness	44	1.0	1.0	49	1.0	1.0	Fading of dots.	+	(-)	c.572G>A, p.Arg191Gln c.927_928insGAA, p.928C>G, :p.L310delinsEV
4 (KA445)	M	Night blindness	37	1.0	1.0	43	1.0	1.0	Changes in location and size of flecks. Fading of dots.	(-)	(-)	c.530T>G, p.Val177Gly c.927_928insGAA, p.928C>G, :p.L310delinsEV
5 (JU0777)	M	Night blindness	59	1.0	0.7	71	0.15	0.2	Fading and disappearance of dots.	+	+	c.927_928insGAA, p.928C>G, :p.L310delinsEV (homo)
6 (JU1255)	M	Night blindness	57	0.8	0.9	65	1.0	1.0	Fading and disappearance of dots.	+	(-)	c.927_928insGAA, p.928C>G, :p.L310delinsEV (homo)
7 (JU1415)	M	Night blindness	58	0.8	1.0	64	0.9	1.0	Fading and disappearance of dots.	NA	+	c.927_928insGAA, p.928C>G, :p.L310delinsEV (homo)
8 (JU0312)	M	Night blindness	3	1.0	1.0	22	1.0	1.0	Changes in location and size of flecks. Conversion from large flecks to small dots.	(-)	(-)	c.530T>G, p.Val177Gly c.927_928insGAA, p.928C>G, :p.L310delinsEV
9 (JU1916)	M	Night blindness	51	1.0	0.6	54	1.0	0.15	NA	NA	+	c.927_928insGAA, p.928C>G, :p.L310delinsEV (homo)
10 (NA1444)	F	Night blindness	61	1.0	1.0	77	1.0	1.0	Fading and disappearance of dots.	+	(-)	c.394G>A, p.Val132Met c.G839A:p.Arg280His
11 (NA0101)	M	Night blindness	14	0.6	0.4	25	1.0	1.0	Changes in location and size of flecks. Conversion from large flecks to small dots.	NA	(-)	c.927_928insGAA, p.928C>G, :p.L310delinsEV (homo)
12 (NA0103)	F	Night blindness	40	1.0	1.0	57	1.0	1.0	Fading and disappearance of dots.	NA	(-)	c.927_928insGAA, p.928C>G, :p.L310delinsEV (homo)
13 (NA0143)	M	Night blindness	59	1.0	1.0	67	1.0	1.0	NA	NA	+	c.G839A:p.Arg280His c.927_928insGAA, p.928C>G, :p.L310delinsEV
14 (KI1001)	F	Night blindness	55	1.0	0.8	66	0.8	1.0	Fading of dots.	+	+	c.927_928insGAA, p.928C>G, :p.L310delinsEV (homo)
15 (KI1064)	F	Night blindness	34	1.0	1.0	55	1.0	1.0	Fading of dots.	+	+	c.712dupG:p.Ala240GlyfsTer19 c.927_928insGAA, p.928C>G, :p.L310delinsEV
16 (KI0080)	F	Night blindness	7	1.0	1.0	15	1.0	1.0	Changes in location and size of flecks. Conversion from large flecks to small dots.	(-)	(-)	c.G839A:p.Arg280His c.927_928insGAA, p.928C>G, :p.L310delinsEV
17 (MI0104)	F	Night blindness	57	1.0	0.9	63	0.8	0.9	Changes in location and size of flecks. Conversion from large flecks to small dots.	NA	(-)	c.927_928insGAA, p.928C>G, :p.L310delinsEV (homo)

BCVA; best-corrected visual acuity, OD; right eye, OS; left eye, OCT; optical coherence tomography, VF; visual field, NA; not available

## Clinical examinations

**Fig. 1:**
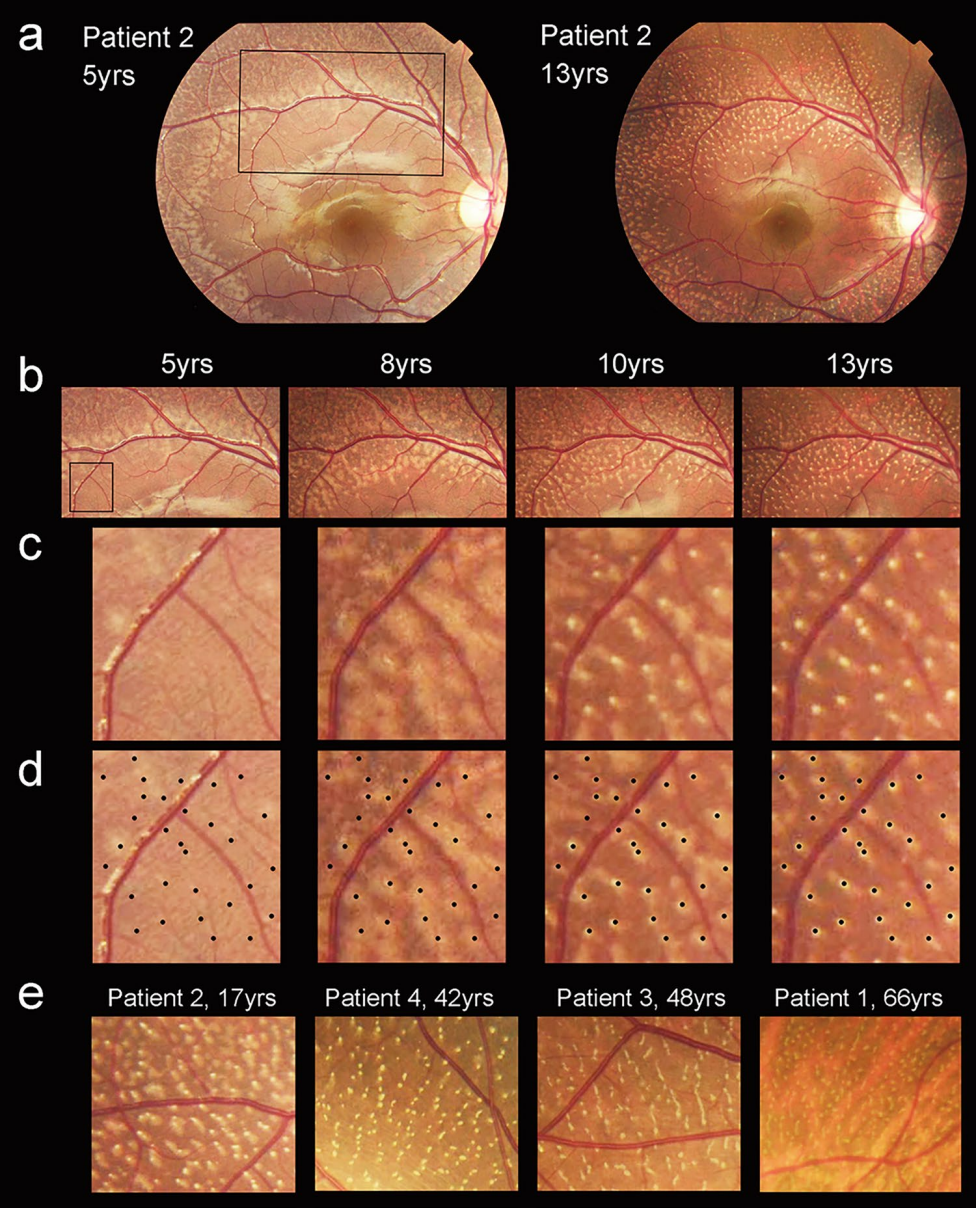
Changes in the appearance of the flecks/dots during the course of the fundus albipunctatus (FA) disease process in Patient 2. **a**: Fundus photographs of the posterior pole at ages 5 and 13 years. **b**. Images of the same region along the superior vascular arcade shown in the rectangle in **a**. The images are presented chronologically from ages 5 to 13 years. **c**. Expanded images of the rectangular region in **b**. **d**. The same images as in **c**, overlayed by the black dots showing the locations of the center of white dots at age 13 years. At age 5 years, the large flecks are sparsely present. At the age 8 years, the flecks are larger and more clearly seen. At age 10 years, the flecks appear to be condensed and smaller. Elongated spots with distinct dots embedded in the peripheral region within each spot can be seen. At age 13 years, the spots appear further compacted and smaller. **e**. The appearance of white dots in the mid-peripheral region in different cases. The size, shape, and intensity differ depending on the patient and the patient’s age.

**Fig. 2:**
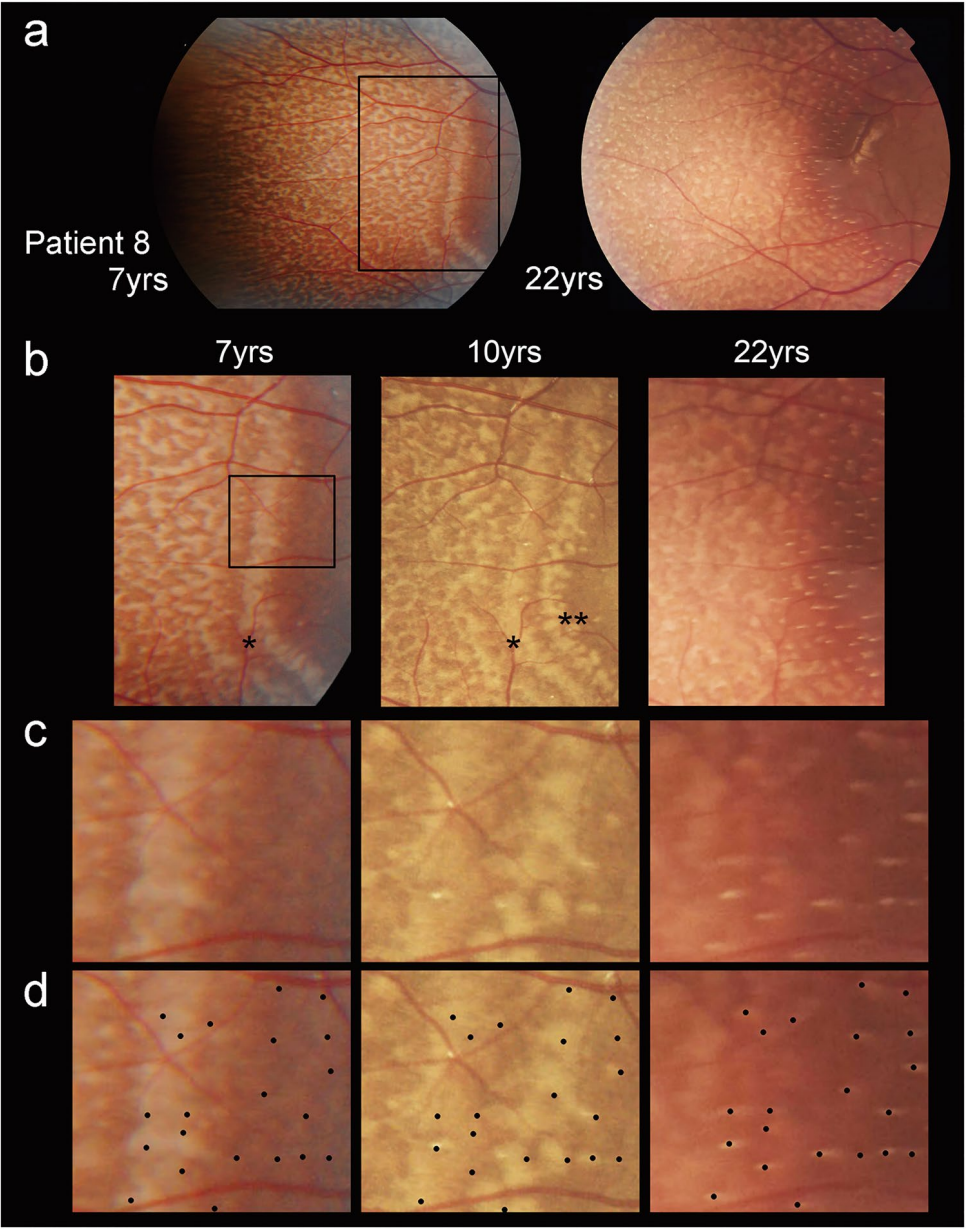
Changes in the appearance of the flecks/dots during the course of the disease process in Patient 8 with FA. **a**. Fundus photographs of the temporal posterior pole at the ages 7 and 22 years. **b**. Chronological images of the same region shown in the rectangle in **a** from ages 7 to 22 years. **c**. Expanded images of the rectangular region in **b**. **d**. The same images as in **c**, overlayed by the black dots showing the locations of the center of the white dots at age 22 years. At age 7 years, there were large flecks scattered in the periphery, and a thick white band (**asterisk in b**) running vertically in the temporal posterior region. At age 10 years, another vertical band appeared (**double asterisks in b**), which is made up of multiple flecks. At the age 22 years, the two bands are not present, and the large flecks are replaced by small and elongated spots or dots scattered in the same locations.

**Fig. 3:**
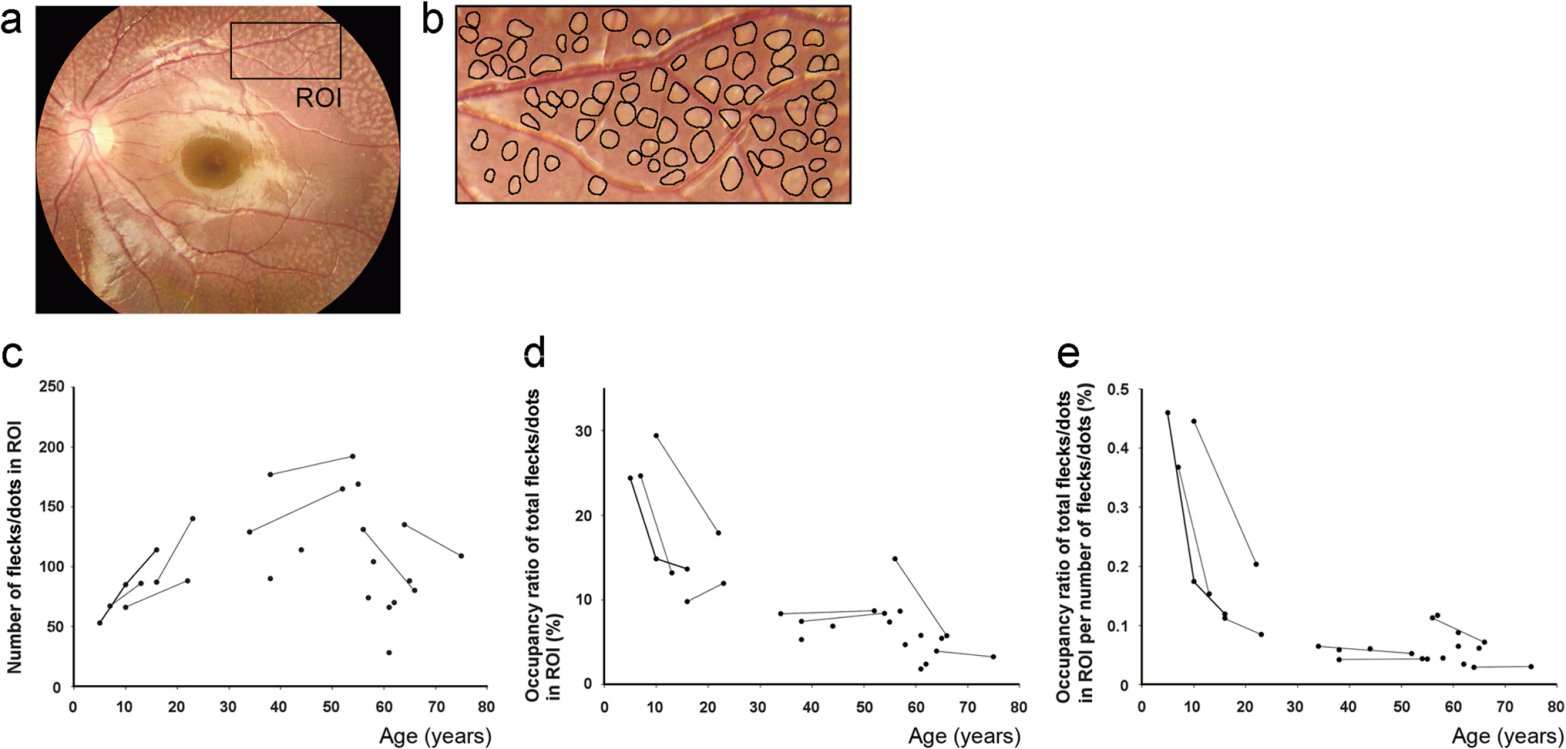
Changes in the number and size of flecks/dots during the disease process. **a**. The region of interest (ROI) is located in the superior-temporal region of the retina along the vascular arcade. **b**. Outlined flecks/dots in the ROI. The large flecks whose outline is located beyond the border of ROI were excluded from the analyses. **c**. Changes in number of flecks/dots in the ROI as a function of the age at the time of examination. The data of the same patients are connected by a solid line. There was a tendency for the number of flecks/dots to increase before the age of 50 years and then decrease thereafter. **d**. Occupancy ratio of total flecks/dots in the ROI. The ratio of retinal area covered by the flecks/dots to the entire ROI was calculated. There was a tendency for the occupancy ratio to decrease during the entire course of the disease progression; rapidly in the first and second decades of life and slowly thereafter. **e**. Occupancy ratio of total flecks/dots in the ROI per number of flecks/dots. This shows the changes in the size of individual flecks/dots. The same tendency was observed as in **d**, but the rapid change during the first and second decades of life can be seen more clearly.

**Fig. 4:**
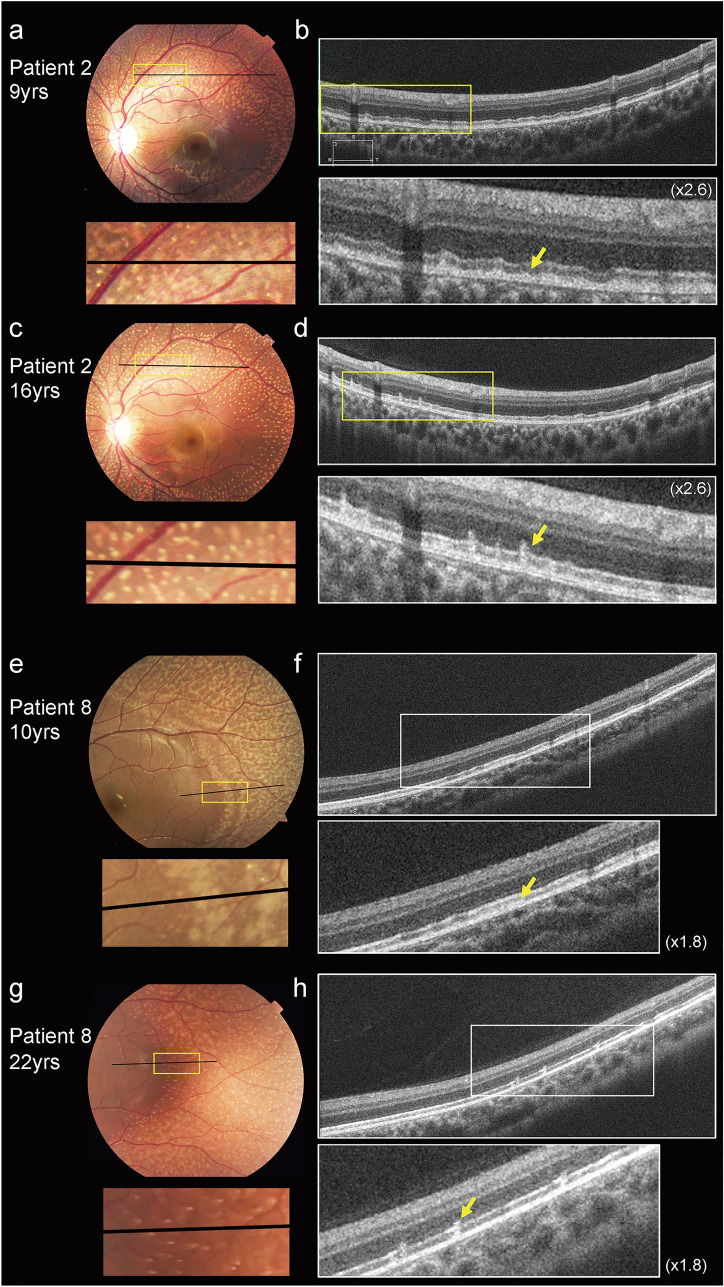
Changes in the fundus photographs and optical coherence tomographic (OCT) images between 9- to 16-years-of-ages in Patient 2 (**a, b, c, and d**), and between 10- to 22-years-of-age in Patient 8 (**e, f, g, and h**). **a**. Fundus photograph of the posterior pole region of Patient 2 at age 9 years. The expanded image showing the location of OCT scan. **b**. OCT image at the location shown in **a**. The expanded image of the nasal region is shown below. At the age 9 years, the large flecks in **a** represent hyperreflective flat deposit on the retinal pigment epithelium (RPE) layer in the OCT image (**arrow**). **c**. Fundus photograph of the same region as in **a** at the age 16 years. The expanded image indicating the location of OCT scan is shown below. At the age 16 years, the flecks have changed to small dots. **d**. OCT image at the location shown in **c**. The expanded image of the nasal region is shown below. The small dots in **c** represent hyperreflective and discrete ‘pile-like formation’. These piles are located between the RPE and external limiting membrane (ELM), and they penetrate into the ellipsoid zone (EZ) of the photoreceptors (**arrow**). Other layer structures, such as the length of photoreceptor outer segments, EZ, and outer nuclear layer (ONL) have not changed. **e**. Fundus photograph of the temporal region of the posterior pole of Patient 8 at the age 10 years. The expanded image showing the location of the OCT scan is shown below. **f**. OCT image at the location shown in **e**. The expanded image of the central region is shown below. At age 10 years, the large flecks in** e** represent hyperreflective flat deposit over the retinal pigment epithelium (RPE) layer in the OCT images (**arrow**). **g**. Fundus photograph of the same region as in **e** at age 22 years. The expanded image showing the location of the OCT scan is shown below. At age 22 years, the flecks have changed to small dots. **h**. OCT image at the location shown in **g**. The expanded image of the central region is shown below. The small dots in **g** represent hyperreflective and discrete ‘pile-like formation’ similar to the findings in Patient 2 (**arrow**).

**Fig. 5:**
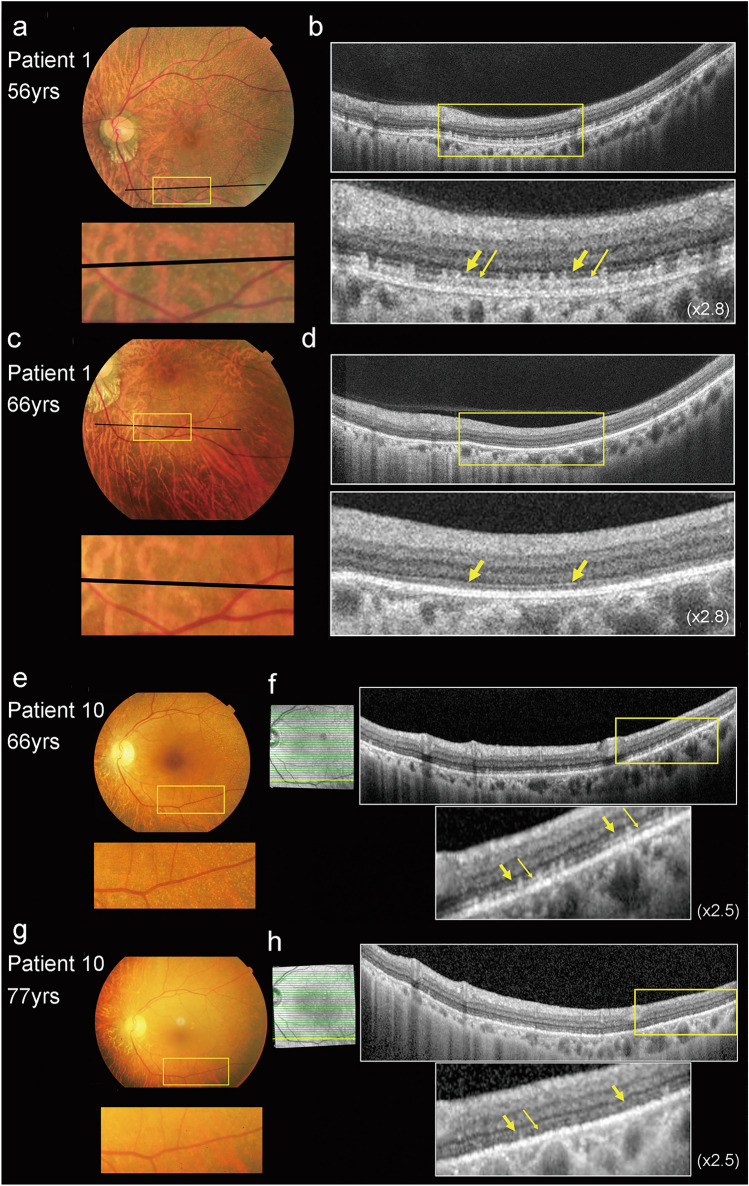
Changes in the fundus photographs and optical coherence tomographic (OCT) images between 56- to 66-years-of-ages in Patient 1 (**a, b, c and d**), and between 66- to 77-years-of-age in Patient 10 (**e, f, g and h**). **a**. Fundus photograph of the posterior pole region of Patient 1 at age 56 years. The expanded image showing the location of OCT scan is shown below. **b**. OCT image at the location shown in **a**. The expanded image of the central region is shown below. At age 56 years, the small dots in **a** represent hyperreflective and discrete ‘pile-like formations’ that are located between the retinal pigment epithelium (RPE) and external limiting membrane (ELM). They penetrate into the ellipsoid zone (EZ) of the photoreceptors. The locations of the ELM and EZ are shown by thick and thin arrows, respectively. **c**. Fundus photograph of the same region as in **a** at the age 66 years. The expanded image showing the location of the OCT scan is shown below. At age 66 years, the white dots are not detected or are less visible than in **a**. **d**. OCT image at the location shown in **c**. The expanded image of the central region is shown below. The pile-like formation could not be observed in the OCT images. The photoreceptor outer segments and EZs are not present. The ELM is located adjacent to the RPE, and the outer nuclear layer (ONL) is thinner than in **b**. The location of the ELM is shown by the thick arrows. **e**. Photograph of the posterior pole region of Patient 10 at age 66 years. The expanded image of the inferior region is shown below. **f**. OCT image at the location shown in **e**. The expanded image of the temporal region is shown below. At age 66, the small dots in **e** represent hyperreflective and discrete ‘pile-like formations’, that are located between the retinal pigment epithelium (RPE) and external limiting membrane (ELM). They penetrate into the ellipsoid zone (EZ) of the photoreceptors. The locations of the ELM or EZ are pointed to by the thick or thin arrows, respectively. **g**. Fundus photograph of the same region as in **e** at age 77 years. The expanded image of the inferior region is shown below. At age 77 years, the white dots are not seen or are less apparent than in **e**. **h**. OCT image at the location shown in **g**. The expanded image of the temporal region is shown below. The pile-like formations are not present in the OCT images. The EZ is less apparent than in **f**. The location of ELM or EZ is shown by the thick or thin arrows, respectively.

Comprehensive ophthalmologic examinations were performed on all cases including measurements of the best-corrected visual acuity (BCVA), plotting of the Goldmann visual fields, ophthalmoscopy, fundus photography (TRC-50DX, Topcon Corporation and CF60UVi, Canon Medtech Supply Corporation; Figs. 1, 2, 3, 4, and 5), spectral-domain optical coherence tomographic (SD-OCT; Cirrus HD OCT, Carl Zeiss Meditec, and Spectralis, Heidelberg Engineering) imaging (Figs. 4 and 5), and electroretinographic (ERG) recordings (LE4000, Tomey Corporation). Full-field ERGs were recorded under both scotopic and photopic conditions in accordance with the standards of the International Society of Clinical Electrophysiology of Vision[10]. The OCT images were consecutively recorded by line scans in the same locations during the course of the disease process. The results of individual patients were compared to follow the long-term morphological changes longitudinally.

### Analysis of white flecks/dots during progression of FA

We determined the number and size of the white flecks/dots in a region of interest (ROI) from the fundus photographs of the left eyes. The ROI was a rectangular area located in the superior-temporal region along the vascular arcade (Figs. 3a and 3b). This region was chosen for the analysis because all of the variations in the appearance of dots/flecks during the disease course, *i.e.*, no fleck, spot-like flecks, elongated spots, and distinct small dots could be observed. Further, the image quality of fundus photographs was better in this region than in the peripheral retina, and suitable for quantitative measurements of the area and number of dots/flecks. The vertical dimension of the ROI was set to match the vertical diameter of the optic disk, and the horizontal width was set to be twice that of the vertical width. Each fleck/dot was manually outlined, and the number of flecks/dots within the ROI and the area (number of pixels within the borders) of the individual fleck/dot were calculated using Image J (1.54 g, NIH; Figs. 3c, 3d, and 3e). To measure the same regions of an individual patient during the course of the disease process, we referred the vascular pattern of superior arcade to match the location of ROIs among the images at different ages (Fig. 3a).

### Genetic analyses

We performed molecular genetic analyses, including the findings of polymerase chain reaction (PCR) with Sanger sequencing and whole exome sequencing, on all patients exhibiting the FA phenotype (Table 1). A detailed explanation of the use of PCR with Sanger sequencing or WES2-5 is reported [8, 11–13]. The frequency of the variants was determined from multiple databases including the Genome Aggregation Database (gnomAD, https://gnomad.broadinstitute.org) and the Japanese Multi Omics Reference Panel (jMorp; https://jmorp.megabank.tohoku.ac.jp/). These databases were also used to identify the variants. To determine whether variants had been reported, searches were conducted in ClinVar (https://www.ncbi.nlm.nih.gov/clinvar/), the Human Gene Mutation Database Professional (HGMD, http://www.hgmd.cf.ac.uk/), and PubMed (https://pubmed.ncbi.nlm.nih.gov/). The pathogenicity of the detected variants was then evaluated based on the criteria set by the American College of Medical Genetics and Genomics.

## Results

The clinical characteristics of the patients are shown in Table 1. The mean ± SD age of the patients at the initial visit (baseline) was 40.9 ± 20.4 years with a range of 3 to 61 years, and at the final visit was 51.6 ± 19.5 years with a range of 15 to 77 years. Night blindness was the initial symptom in all patients. The average ± SD BCVA at the baseline was 0.025 ± 0.058 logarithm of the minimum angle of resolution (logMAR) units OD and 0.057 ± 0.106 logMAR units OS. At the final visit, the BCVA was 0.062 ± 0.187 logMAR units OD and 0.092 ± 0.245 logMAR units OS. Although there were 7 cases that developed macular degeneration during the follow-up period, the average BCVA was not significantly different between the baseline and the final visit (Student *t* tests; *P* = 0.24, OD; and *P* = 0.26, OS).

### Longitudinal changes in the appearance of the flecks/dots

The fundus photographs of patient 2 taken at the same retinal location between ages 5 and 13 years are arranged chronologically in Figures 1b and 1c. At age 5 years, the large flecks were sparsely distributed near the vascular arcade **(**Figs. 1b and 1c, 5** yrs)**. At age 8 years, the flecks were larger and more clearly seen **(**Figs. 1b and 1c, 8** yrs)**. At age 10 years, the flecks appeared compressed and smaller, and elongated spots with distinct dots embedded in the peripheral region of each spot were present. **(Fig. Figs.** 1**b and 1c, 10 yrs)**. At the last visit at age 13 years, the spots were further compressed, and distinct dots were also present throughout the ROI **(Figs.** 1**b and 1c, 13 yrs).**

The fundus photographs of patient 8 taken at the same location between ages 7 and 22 years showed similar changes during the course of the disease process **(**Fig. 2**)**.

In 13 of the 15 other cases, there were significant changes in the flecks/spots during the progression of the disease process. The changes were in the location of flecks/dots, changes of large flecks to small dots, and the fading or disappearance of the dots at the older age (Table 1).

### Changes in the number and size of the flecks/dots

We counted the number and relative size of the flecks/dots in the ROI in all cases **(**Fig. 3**)**. The number of flecks/dots in the ROI are plotted relative to the age at the time the photographs were taken. The data of the same patient are connected by a solid line **(**Fig. 3c**)**. There was a tendency for the number of flecks/dots to increase during the course of the disease process before the age of 50 years and then decrease.

To estimate the changes in the size of flecks/dots during the disease process, the ratio of the retinal area covered by the flecks/dots to the entire ROI was calculated and designated as the ‘occupancy ratio’ of total flecks/dots in the ROI **(**Fig. 3d**)**. There was a tendency for the occupancy ratio to decrease during the entire course of the disease process; rapidly in the first and second decades of life and slowly thereafter.

We also calculated the ‘occupancy ratio of the total flecks/dots in the ROI per number of flecks/dots’ to determine the changes in the size of individual flecks/dots **(**Fig. 3e**)**. The rapid change during the first and second decades of life was detected more clearly than in Figure 3d**.**

### Three-dimensional changes of the flecks and dots

We further examined the three-dimensional changes of the flecks and dots by comparing the OCT images taken at the same location of the posterior retina during first to third decades of life **(Patients 2 and 8, **Fig. 4**)** and sixth to eighth decades of life **(Patients 1 and 10, **Fig.5**)**.

In Patient 2, the large flecks observed at age 9 years represented hyperreflective flat deposit over the RPE layer in the OCT images **(**Figs. 4a and 4b**)**. At age 16 years, the flecks had changed to small dots **(**Fig. 4c**)**, and the flat deposits over the RPE in the OCT image changed to hyperreflective and discrete ‘pile-like formations’. They were located between the RPE and ELM and extended into the EZ **(**Fig. 4d**)**.

Other layered structures, such as the length of the photoreceptor outer segments, EZs, and outer nuclear layer (ONL) did not change during this period.

The OCT images of patient 8 taken at the same location between ages 10 and 22 years showed similar changes during the course of the disease process **(**Figs. 4e, 4f, 4g and 4h**)**.

In Patient 1, small dots were present next to the inferior vascular arcade, and they corresponded to the pile-like formations in the OCT images at age 56 years **(**Figs. 5a and 5b**)**. Ten years later at age 66 years, the white dots were not present or had become less visible **(**Fig. 5c**).** In addition, the pile-like formations could not be observed in the OCT images **(**Fig. 5d**)**. It is noteworthy that the atrophy of photoreceptor outer segments was observed at 66-years-of-age. The EZs were not present, and the external limiting membrane (ELM) was located next to the RPE, and the ONL was thinner. The distance between the ELM and anterior edge of the RPE was less than one-half that at age 56 years. Atrophy of photoreceptor layer was also observed in Case 10 during the follow-up period from 66- to 77-years-of-age **(**Figs. 5e, 5f, 5g and 5h**)**.

### Changes in peripheral visual field

**Fig. 6:**
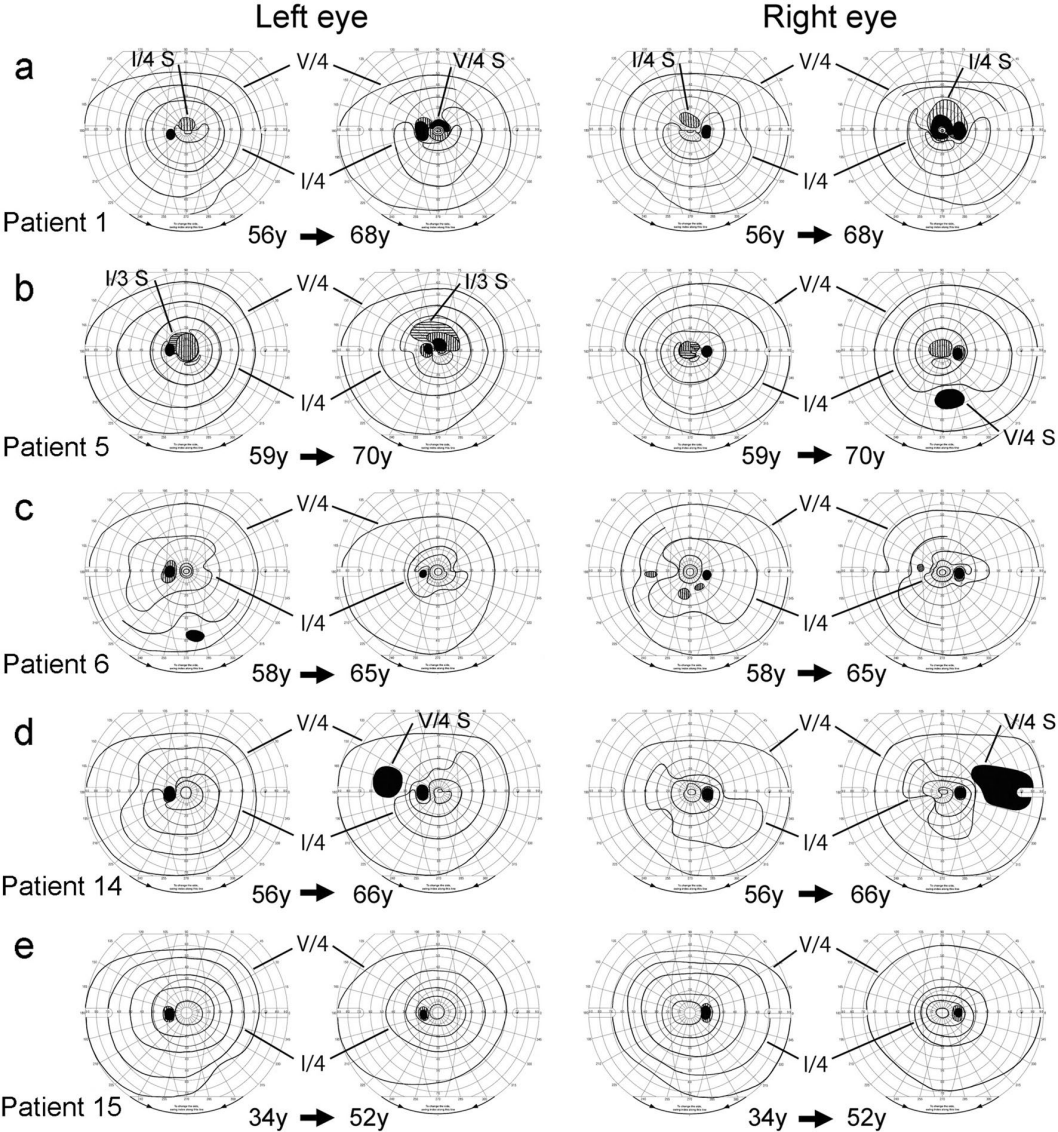
Changes in visual field during the course of the disease process determined by Goldmann perimetry. Changes in the visual field are shown in 5 representative cases, with intervals between 7 to 18 years. In all the cases, there was a peripheral constriction of visual field as indicated by the changes of the I/4 isoptor. Additionally, scotomas emerged or expanded in the midperipheral or paracentral regions in 3 cases **(a, b, and d)**

To determine the changes in the function of the peripheral photoreceptors, we compared the visual fields obtained by Goldmann perimetry during the course of the disease process in 5 representative cases, with intervals between 7 to 18 years **(**Fig. 6**)**. In all cases, there appeared to be a constriction of the peripheral visual fields as indicated by the changes of the I/4 isoptor. Additionally, scotomas emerged or expanded in the midperipheral or paracentral regions in 3 cases **(**Figs. 6a, 6b, and 6d**).**

**Fig. 7:**
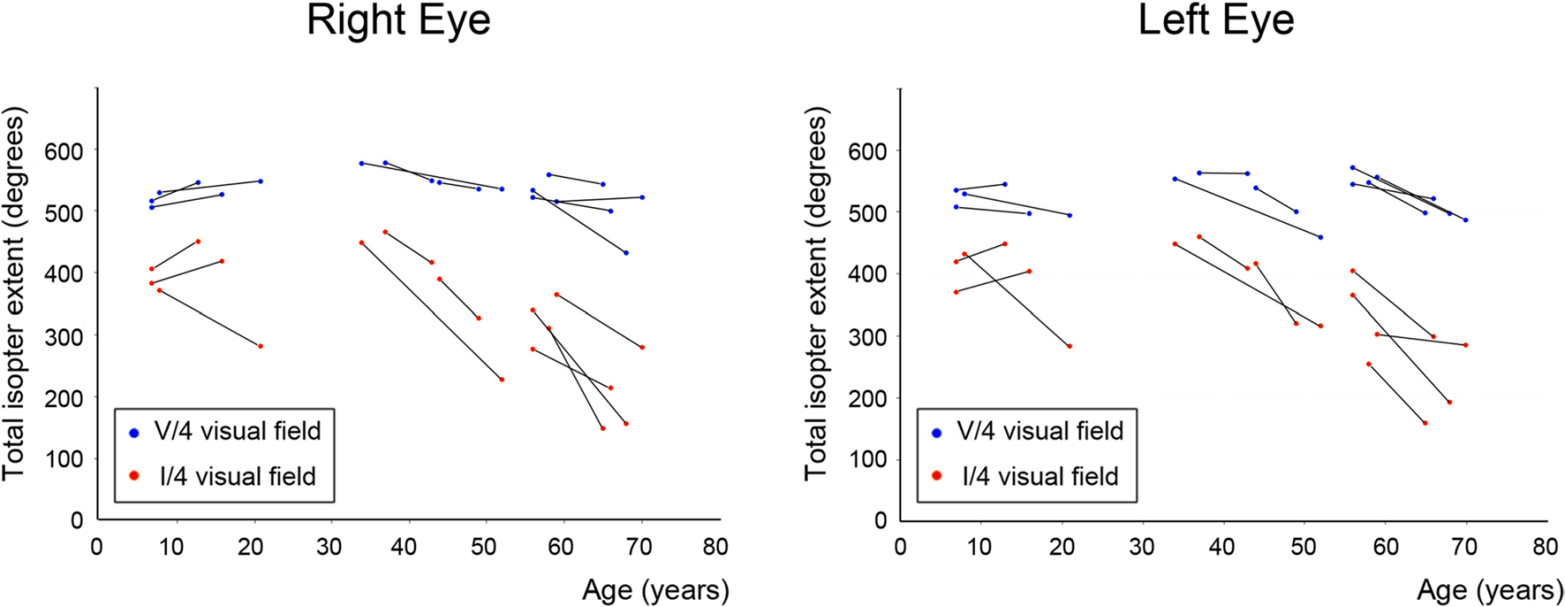
Longitudinal changes in the extent of the total visual field during the follow-up period. The extent of V/4 and I/4 isoptors (degrees) were summed in 8 directions to represent the total visual field extent. The results of I/4 isoptors (red marks) indicated that the constriction of the peripheral visual field was apparent at ages over 40 years in both eyes.

We calculated the longitudinal changes of the peripheral visual field by Goldmann kinetic visual field test in 10 patients where the visual fields could be compared with intervals of over 5 years **(**Table 1**, **Fig 7**)**. For the analysis, the extent of V/4 and I/4 isoptors (degrees) were summed in 8 directions to represent the total visual field extent. The results for the I/4 isoptors clearly indicated that constriction of peripheral visual field was apparent at ages over 40 years **(**Table 1**, **Fig 7**)**.

## Discussion

We have investigated the longitudinal changes of the morphology of the retina and the overall function of the visual system in patients with FA by detailed analyses of the fundus photographs, OCT images, BCVA, and Goldmann visual fields.

The examinations of the fundus photographs showed that spot-like or irregularly-shaped flecks were present in the peripheral retina in the first decade of life **(**Figs. 1c, 5** yrs and 8 yrs)**. Then the flecks became smaller, and elongated spots with distinct dots embedded at the peripheral edge appeared in the second decade of life **(Fig.** 1**c, 10 and 13 yrs)**. These elongated spots became smaller in the middle age **(Fig. **5**a, 56 yrs)**. Finally, part of the dots decreased in intensity or disappeared at ages > 60-years-of-age **(Fig.** 5**c, 66 yrs)**.

The number of flecks and dots increased during the disease process before the age of 50 years, and then the number decreased **(**Fig. 3c**)**. The size of the individual flecks/dots also decreased rapidly during the first and second decades of life, and the rate of decrease became slower thereafter **(**Fig. 3e**)**.

**Fig. 8:**
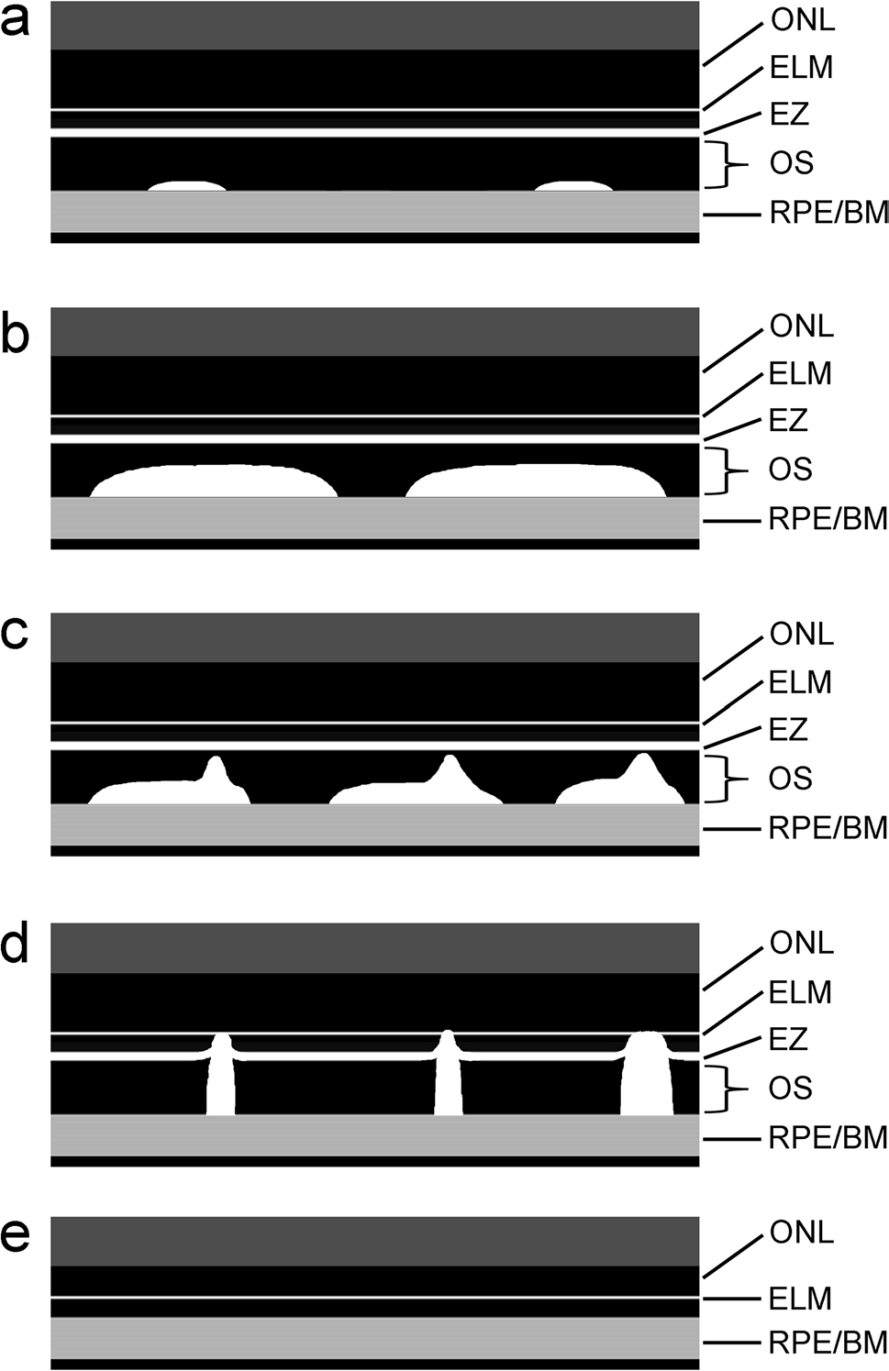
The natural course of the flecks/dots in eyes with fundus albipunctatus. The morphological changes of the flecks/dot observed by optical coherence tomography (OCT) is schematically presented from **a** to **e**. **a**. The flecks in the first decade of life are observed as hyperreflective flat deposits over the RPE layer. **b**. The location and size of the deposits in **a** vary during childhood and adolescence. **c**. In the second decade of life, small protrusions are present at the peripheral part of individual deposits which expand toward the ellipsoid zone (EZ) of the photoreceptors. **d**. The flat deposits become smaller, and only the protrusions are left that compose the hyperreflective ‘pile-like formation’ that penetrate the EZ toward the external limiting membrane (ELM). This ‘pile-like formation’ can be observed from the second to sixth decade of life. **e**. In the seventh decade of life, the pile-like formations are less apparent or not present and are accompanied by the loss of the EZ and shortening of the photoreceptor outer segments. Due to the shortening of the outer segments, the ELM is located adjacent to the retinal pigment epithelium (RPE), and the outer nuclear layer (ONL) appears thinner than in normal eyes.

The natural course of the flecks/dots in FA is presented schematically from the longitudinal observations of the OCT images **(**Fig. 8**)**. In the first decade of life, the large flecks were observed as hyperreflective flat deposits over the RPE layer **(**Figs. 8a and 8b**)**. In the second decade of life, small protrusions emerged in the peripheral part of the individual deposits which extended toward the EZ of the photoreceptors **(**Fig. 8c**)**. With increasing age, the flat deposits became smaller, and only the protrusions remained to make up the hyperreflective pile-like formation which penetrated into the EZ towards the ELM **(**Fig. 8d**).** This progression could be observed until the sixth decade of life. After the seventh decade of life, the ‘pile-like formations’ disappeared together with a loss of the EZ and shortening of the photoreceptor outer segments **(**Fig. 8e**)**. The atrophy of photoreceptor layer was the most likely cause of the peripheral visual field defects in the later stages **(**Figs. 6 and 7**)**.

The morphological features of the white dots are described by several studies with a small number of the cases. Marmor describes some characteristic features of the white dots; the long-term follow-up of 13 to 14 years revealed that there was an evolution in the appearance from flecks to dots and an increase in number of dots during aging [14]. On the other hand, Nakamura et al report that the number ofwhite dots decreased with age and were not detected in older patients [6]. The findings in the earlier studies are systematically confirmed in this study **(**Fig. 3**)**, and we have further determined the correlations between the morphological changes of the white dots and the physiological function. Our analyses of the OCT images indicates that the mid-peripheral visual field defects in older age can be attributed to the atrophy of the photoreceptor layer at the same locations **(**Fig. 5**)**.

Niwa et al. report that 28.6% (4/14) of the patients with FA had abnormally reduced rod b-wave amplitudes even after prolonged dark-adaptation of 180 minutes. This indicates that not only the cone, but also the rod system was irreversibly dysfunctional in patients with FA. Although macular degeneration in FA is reported in many publications [5–8], there are no reports that show an apparent photoreceptor atrophy in the peripheral or mid-peripheral retina of the patients. Considering that the *RDH5* gene plays an important role in the rod visual cycle, it is reasonable that there is a progressive atrophy of the rod photoreceptors in older patients together with preexisting macular degeneration or cone dysfunction.[15]

The pile-like formations in the OCT images in FA are described in other studies, [16–22] but there are no longitudinal observations to demonstrate the natural course of the white dots. We have clearly shown the following stages; 1) hyperreflective flat deposits over the RPE layer **(**Figs. 8a and 8b**)**, 2) hyperreflective flat deposits with small protrusions **(**Fig. 8c**)**, 3) hyperreflective pile-like formation penetrating the EZ toward the ELM **(**Fig. 8d**)**, and 4) disappearance of the pile-like formations accompanied by the loss of the EZ and shortening of the photoreceptor outer segments **(**Fig. 8e**)**. These morphological changes suggest that the early and transformable deposits become condensed or reduced in the early stage, and move into the narrow spaces between the photoreceptor outer segments. Alternatively, the deposit can move upward into the space where photoreceptor outer segments are shortened [23]. Due to the barrier-function of the ELM, the deposits stop expanding below the ELM and remain at the same location for a long period of time.

Similar OCT findings of the white dots in the peripheral retina have been observed in other retinal diseases. In the visual cycle-related retinal dystrophies with white-dot lesions, such as *RLBP1*-related retinopathy [21], *RPE65*-related retinopathy,[24] and *LRAT*-related retinopathy,[25] the hyperreflective pile-like formations between the ELM and RPE could be observed. In the retinopathy due to Vitamin A deficiency [26, 27], both the deposit-like hyperreflective material on the RPE and the hyperreflective pile-like formation between the ELM and RPE have been observed. In reticular pseudodrusen [23, 28–30] and the diffuse-trickling type of geographic atrophy [31, 32], hyperreflective pile-like formations arising from the RPE can be observed in the location of the subretinal deposits.

The exact composition of the deposit in eyes with FA has not been determined. Possibly, cis-retinols and cis-retinyl esters in the RPE may accumulate due to the lack of activity in 11-cys-retinol dehydrogenase [33]. The protein products of the *RDH5*, *RPE65*, *LRAT,* and *RLBP1* are located in the RPE and function cooperatively in the visual cycle for the regeneration of 11-cys-retinal. The components of the deposit observed in these genetic disorders and vitamin A deficiency may be common to some extent. These visual cycle disorders share the characteristic feature of not only the morphological structures of the deposit, but also the short-wavelength fundus autofluorescence where decreased autofluorescence is observed homogeneously in the peripheral retina [17, 21, 34]. Thus, it was not unexpected that patients with *RDH5*-related FA in the late-stage show peripheral photoreceptor atrophy and reduced scotopic ERG function[15], which appeared in the earlier stages of *RPE65*, *LRAT,* and *RLBP1*-related retinopathies.

The question remains as to why macular degeneration and cone dysfunction are reported as more prominent than peripheral photoreceptor atrophy in patients with FA. We do not have a definitive answer; however, dysfunction of the rod visual cycle may play a significant role in the development of macular atrophy and cone dysfunction that emerge in a subset of patients before middle age.

The limitation of our study is that there was not a single case where the entire life-time morphological changes of flecks/dots could be observed. However, due to the nature of this disease, it is necessary to follow the same patients for over 50 years to observe the lifetime profile of the flecks/dots. Thus, cross-sectional analysis of different cases was also valuable in revealing the natural course of the flecks/dots.

The clinical stages of OCT features during disease progression have been schematically proposed (Fig. 8). However, these stages were not determined quantitatively but were based on qualitative observations of representative cases. To achieve a better understanding of the etiology of this disease, quantitative analyses of OCT images in a large cohort of patients will be necessary, and this will be a focus of our future investigations.

In conclusion, we present detailed long-term morphological changes of the peripheral flecks/dots during the course of the disease process in patients with FA. The atrophy of the photoreceptor layer in the peripheral retina appears to be strongly related to the peripheral visual field defects in the advanced stage. Thus, FA has been found to be a slowly progressive retinal dystrophy involving atrophy of the rod photoreceptors in older patients. This finding should provide important information for the patients during genetic counseling for this disease because knowing the likelihood of the appearance of peripheral visual field defect in old age is important in considering patients' life plans. Further, the etiology commonly observed in the visual cycle-related diseases and other acquired age-related diseases[28, 31, 32] may provide keys for the treatment of these disorders.
